# Supplementary figures and images for: Awareness of security and privacy settings in video conferencing apps among faculty during the COVID-19 pandemic
Source: PeerJ Comput Sci. 2022 Jul 7;8:e1021. doi: 10.7717/peerj-cs.1021 (PMC9299235; doi:10.7717/peerj-cs.1021)

**Appendix 1**

**
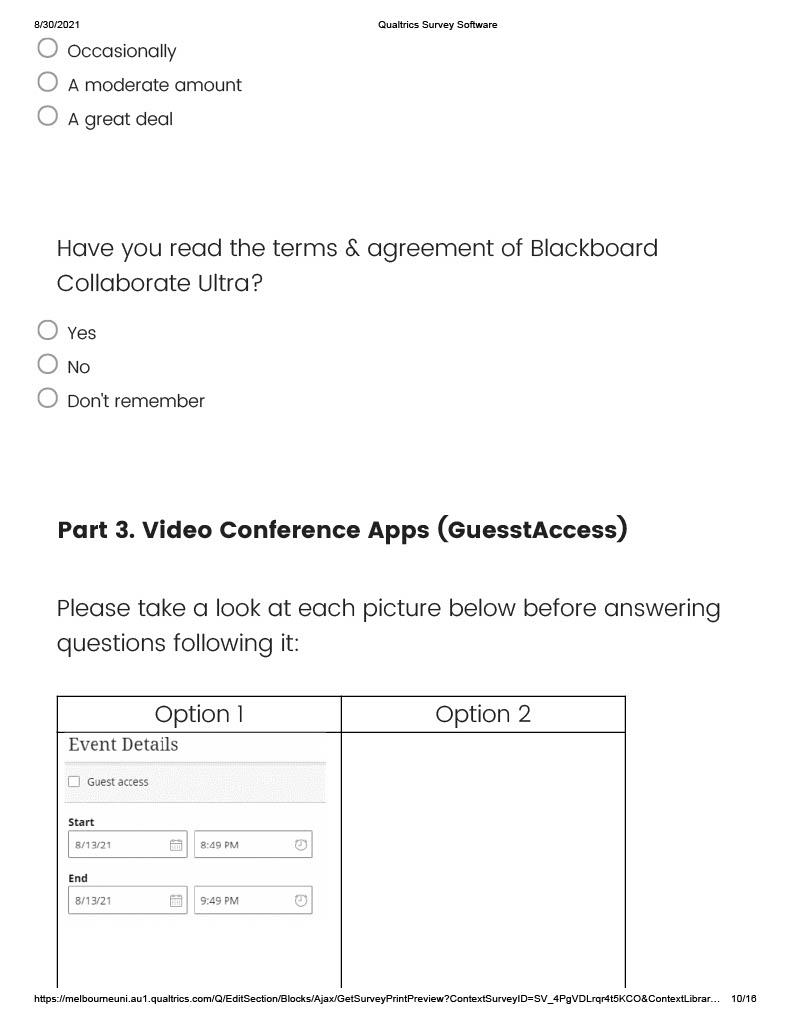
**

**
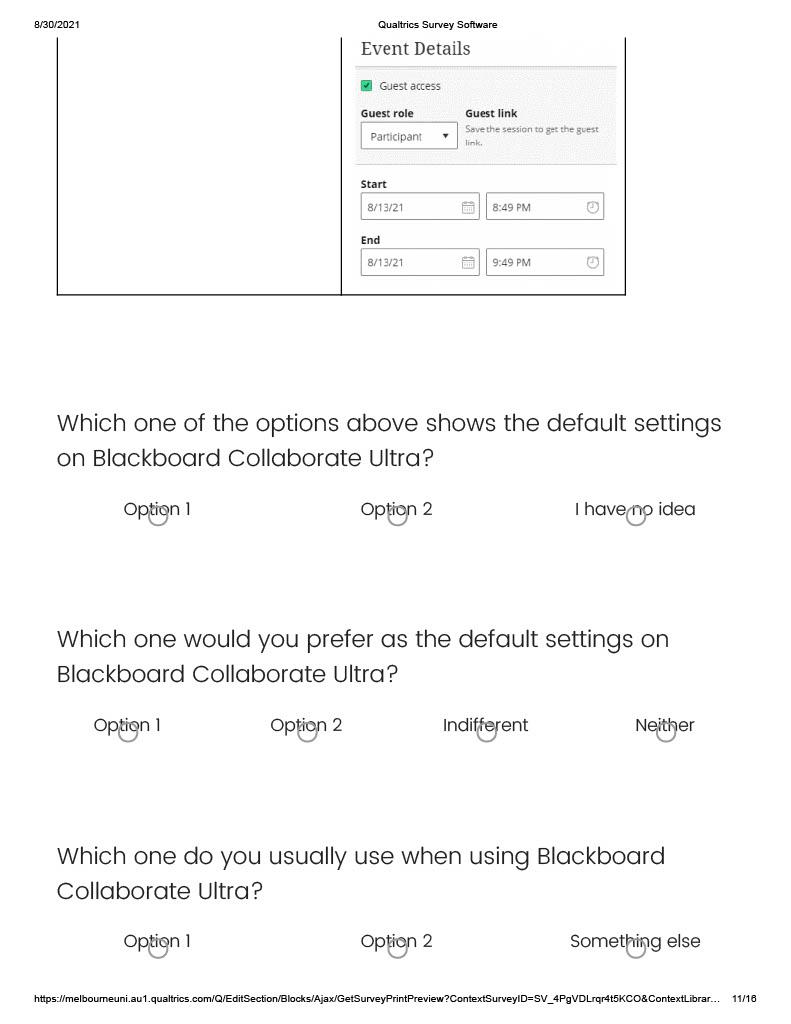
**

**
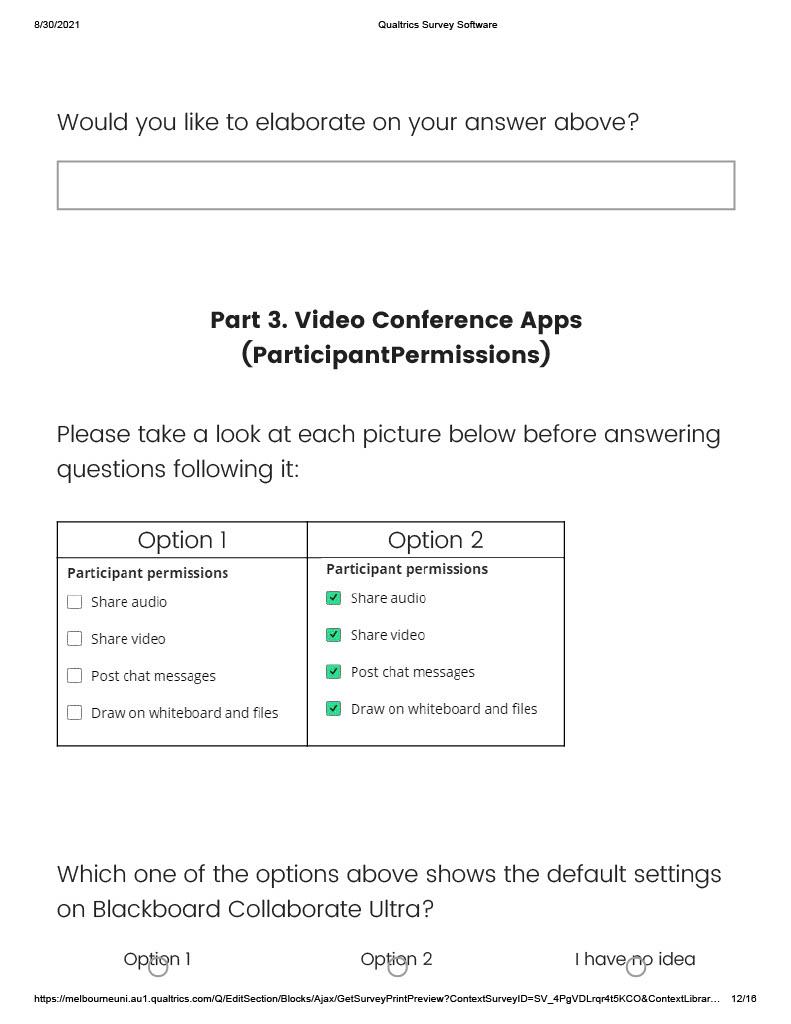
**

**
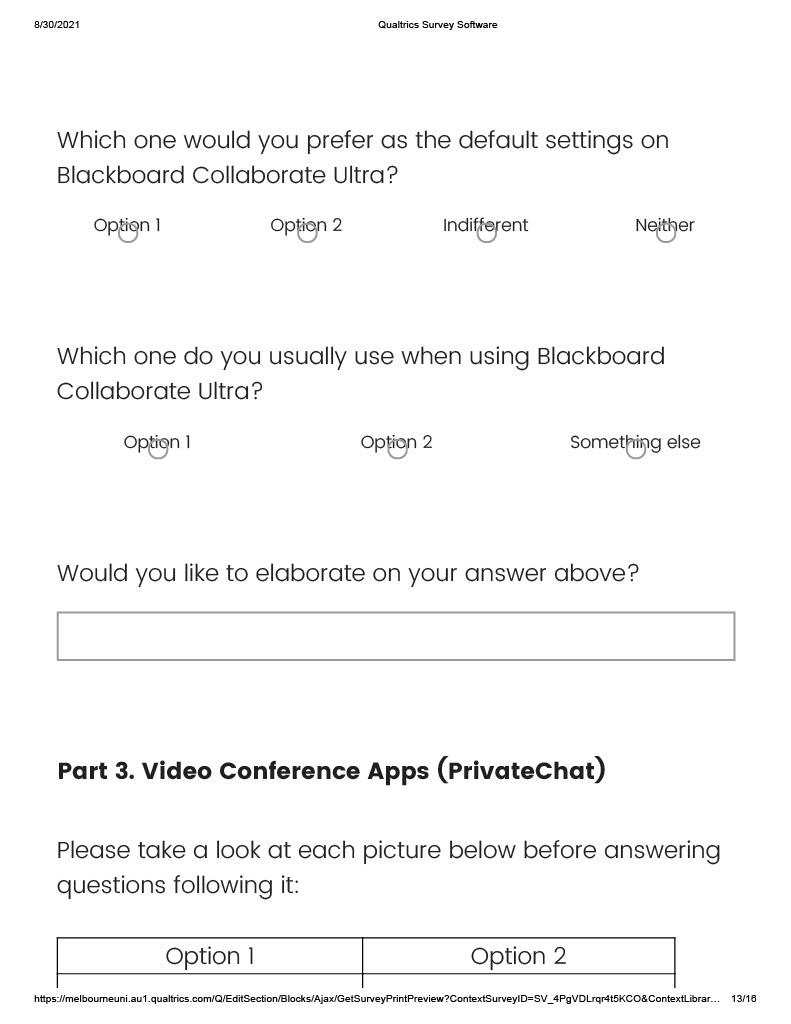
**

**
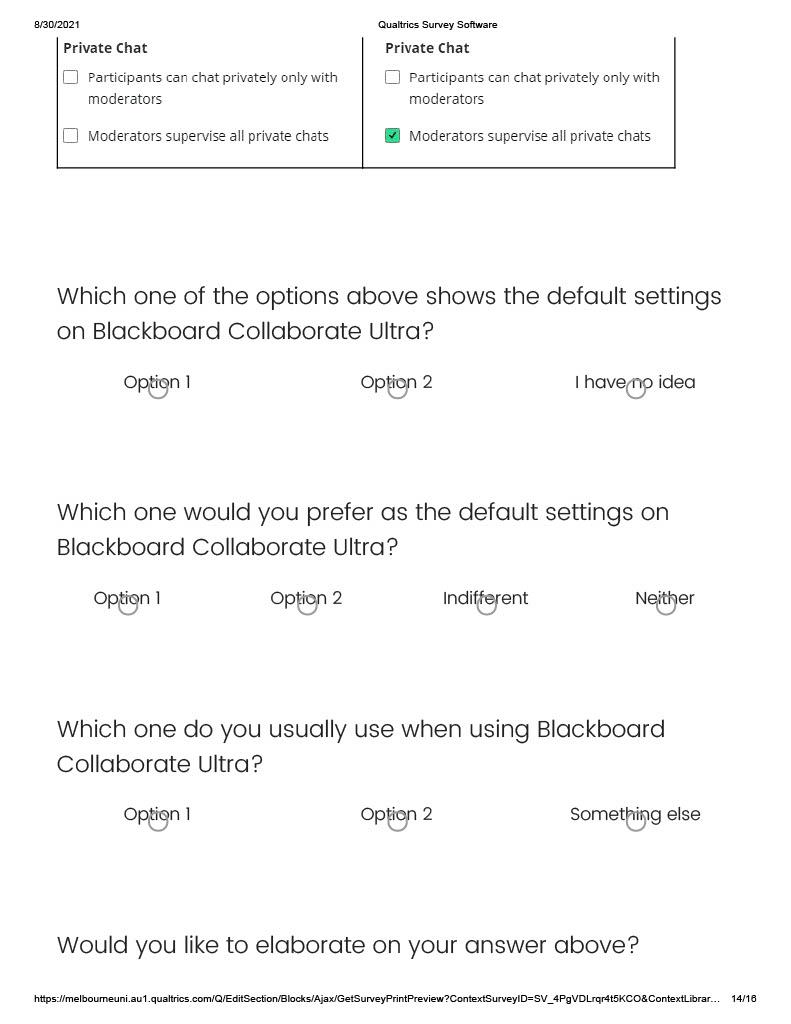
**

**
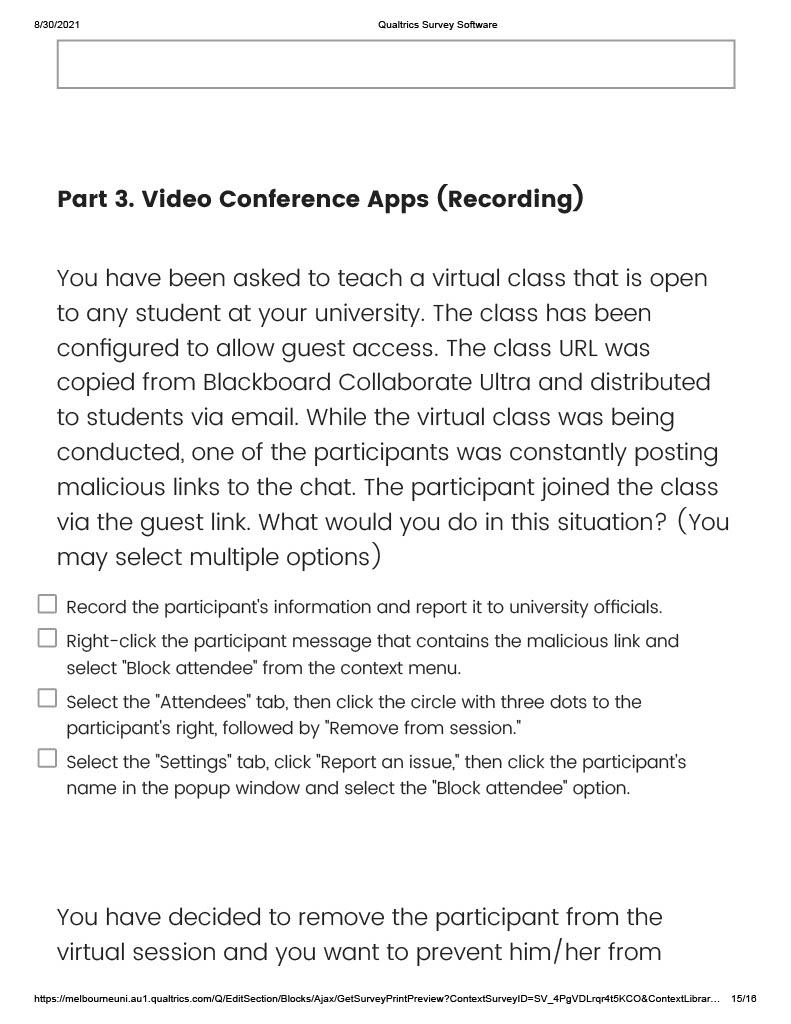
**

**
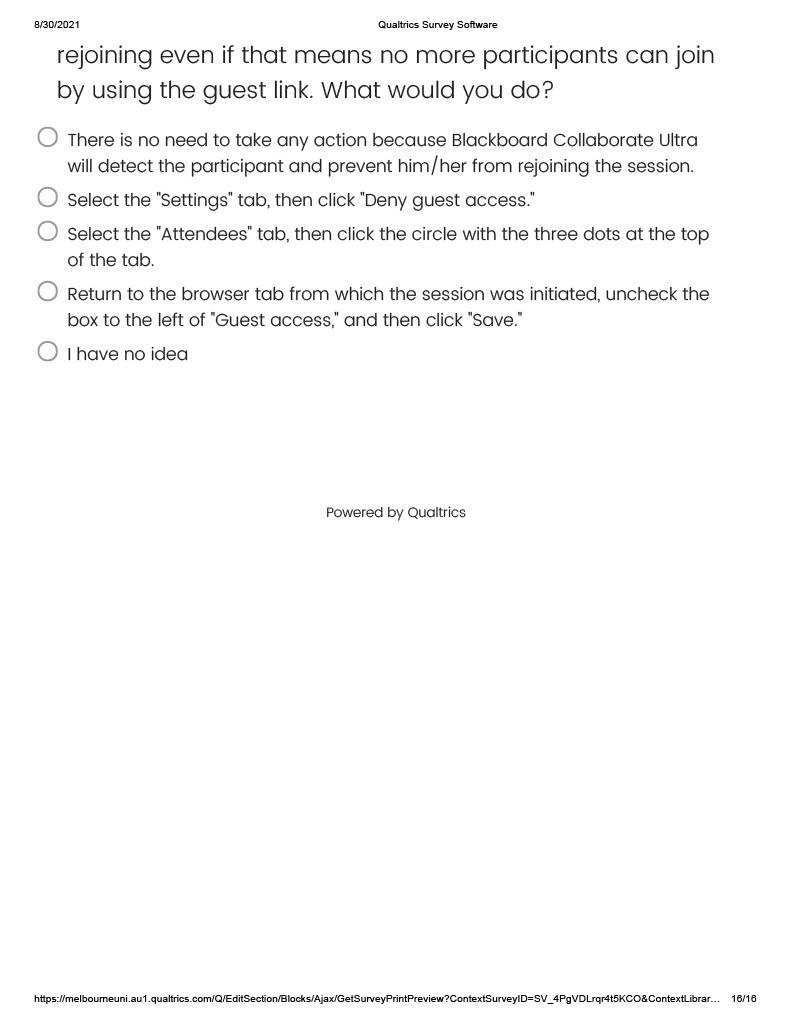
**

Supplement: Supplemental Information 2 [file peerj-cs-08-1021-s002.docx]
