# Supplementary material for: Awareness of security and privacy settings in video conferencing apps among faculty during the COVID-19 pandemic
Source: PeerJ Comput Sci. 2022 Jul 7;8:e1021. doi: 10.7717/peerj-cs.1021 (PMC9299235; doi:10.7717/peerj-cs.1021)
Supplement: Supplemental Information 3 [file peerj-cs-08-1021-s003.pdf]

var	description	0	1	2	3	4	5
gender	What is your gender?	Male	Female				
age	What is your age?		25 - 34	35 - 44	45 - 54	55 - 64	
edu	What is your level of education?	Bachelor	Master	PhD			
rank	What's your Academic level/rank?	Teaching assistant	Lecturer	Assistant Professor	Associate Professor	Full Professor	
exp	How many years of teaching experience do you have?		< 3 years	3 to 9 years	10 to 24 years	25 +	
field	What is your college area of specialization?		Art, Humanities and Social Sciences	Science, Engineering and Technology	Medical and Health Sciences		
att1	Attitudes - I like using ICT for teaching or conducting research		Stongly Disagree	Disagree	Neutral	Agree	Strongly Agree
att2	Attitudes - ICT makes teaching or conducting research more interesting		Stongly Disagree	Disagree	Neutral	Agree	Strongly Agree
att3	Attitudes - I am more motivated to teach or to conduct research with ICT		Stongly Disagree	Disagree	Neutral	Agree	Strongly Agree
att4	Attitudes - There is a lot of potential in the use of mobile technologies (e.g. smartphones, tablets) for teaching and research		Stongly Disagree	Disagree	Neutral	Agree	Strongly Agree
dl1	Digital Literacy - I know how to solve my own technical problems.		Stongly Disagree	Disagree	Neutral	Agree	Strongly Agree
dl2	Digital Literacy - I can learn new technologies easily.		Stongly Disagree	Disagree	Neutral	Agree	Strongly Agree
dl3	Digital Literacy - I know about a lot of different technologies.		Stongly Disagree	Disagree	Neutral	Agree	Strongly Agree
dl4	Digital Literacy - I am confident with my search and evaluate skills in regards to obtaining information from the Web		Stongly Disagree	Disagree	Neutral	Agree	Strongly Agree
dl5	Digital Literacy - I am familiar with issues related to web-based activities e.g. cyber safety, search issues, plagiarism		Stongly Disagree	Disagree	Neutral	Agree	Strongly Agree
dl6	Digital Literacy - ICT enables me to collaborate better with their peers on project work and other learning activities		Stongly Disagree	Disagree	Neutral	Agree	Strongly Agree
dl7	Digital Literacy - I frequently obtain help with my university work from my friends over the Internet e.g. through Email, Social Media, or Videoconference		Stongly Disagree	Disagree	Neutral	Agree	Strongly Agree
pc1	Privacy Concerns - User online privacy is really a matter of users' right to exercise control and autonomy over decisions about how their information is collected, used, and shared.		Stongly Disagree	Disagree	Neutral	Agree	Strongly Agree
pc2	Privacy Concerns - I believe that online privacy is invaded when control is lost or unwillingly reduced as a result of a marketing transaction.		Stongly Disagree	Disagree	Neutral	Agree	Strongly Agree
pc3	Privacy Concerns - Companies seeking information online should disclose the way the data are collected, processed, and used.		Stongly Disagree	Disagree	Neutral	Agree	Strongly Agree
pc4	Privacy Concerns - It is very important to me that I am aware and knowledgeable about how my personal information will be used.		Stongly Disagree	Disagree	Neutral	Agree	Strongly Agree
pc5	Privacy Concerns - When online companies ask me for personal information, I sometimes think twice before providing it.		Stongly Disagree	Disagree	Neutral	Agree	Strongly Agree
pc6	Privacy Concerns - I'm concerned that online companies are collecting too much personal information about me.		Stongly Disagree	Disagree	Neutral	Agree	Strongly Agree
sa1	Security Awareness - Overall, I am aware of the potential security threats and their negative consequences.		Stongly Disagree	Disagree	Neutral	Agree	Strongly Agree
sa2	Security Awareness - I have sufficient knowledge about the cost of potential security problems.		Stongly Disagree	Disagree	Neutral	Agree	Strongly Agree
sa3	Security Awareness - I understand the concerns regarding information security and the risks they pose in general.		Stongly Disagree	Disagree	Neutral	Agree	Strongly Agree
sa4	Security Awareness - I know the rules and regulations prescribed by the security policy of my university.		Stongly Disagree	Disagree	Neutral	Agree	Strongly Agree
sa5	Security Awareness - I understand the rules and regulations prescribed by the security policy of my university.		Stongly Disagree	Disagree	Neutral	Agree	Strongly Agree
sa6	Security Awareness - I know my responsibilities as prescribed in the security policy to enhance the information security of my university.		Stongly Disagree	Disagree	Neutral	Agree	Strongly Agree
fam1	How familiar are you with Blackboard Collaborate Ultra?		Not at all familiar	Slightly familiar	Moderately familiar	Somewhat familiar	Extremely familiar
fam2	How much do you use Blackboard Collaborate Ultra?		Never	Rarely	Occasionally	A moderate amount	A great deal
toa	Have you read the terms & agreement of Blackboard Collaborate Ultra?	No	Yes	Don't Remember			
